# Are interventional radiology and allied specialities neglected in undergraduate medical education? A systematic review

**DOI:** 10.1016/j.amsu.2019.03.004

**Published:** 2019-03-15

**Authors:** Elif Iliria Emin, Zeinab Ruhomauly, Iakovos Theodoulou, John Gerrard Hanrahan, Nikolaos Staikoglou, Marios Nicolaides, Narayanan Thulasidasan, Apostolos Papalois, Michail Sideris

**Affiliations:** a Faculty of Life Sciences and Medicine, King's College London, London, UK; b Medical School of Aristotle University of Thessaloniki, Thessaloniki, Greece; c Barts and the London School of Medicine and Dentistry, Queen Mary University of London, London, UK; d Guys and St Thomas' Hospital, King's College London, London, UK; e Experimental Educational and Research Centre ELPEN, Athens, Greece

**Keywords:** Interventional radiology, Interventional cardiology, Systematic review, Medical education, Simulation-based learning

## Abstract

**Objectives:**

Minimally invasive interventional approaches are gaining wider acceptance with several specialities incorporating such principles. Awareness and understanding of interventional principles require efficacious education and training methodologies. We performed a systematic review to identify all available interventional speciality learning modules or training opportunities available for undergraduate medical students. We also propose a standardised framework for relevant modules.

**Methods:**

We searched PubMed and all Ovid databases with no language restriction for studies that report and evaluate interventional speciality educational modules or similar training initiatives. We followed a prospective protocol (PROSPERO registration: CRD42018110006). Internal and external validity of the included studies was assessed. Qualitative synthesis of data was performed to define performance improvement and/or motivation towards a career in an interventional speciality.

**Results:**

Out of 6081 records, 17 studies met the inclusion criteria, 15 of which were focused on interventional radiology. More than half of studies (9/17) were surveys where student knowledge and interest were reported as poor. 5 out of 6 studies which assessed the effect of educational interventions concluded to improved knowledge or performance. Most surveys concluded that early exposure can increase interest towards such specialities, improve knowledge and relevant motivation.

**Conclusions:**

Few studies report teaching initiatives in interventional radiology and other interventional specialities, reflecting the poor relevant motivation and knowledge amongst medical students. Simple interventions e.g. introductory lectures and simulation sessions spark interest in students and also improve knowledge as proven in the case of interventional radiology. Standardisation of such efforts via a suggested framework, Strategy Development Framework for Interventional Radiology, can further optimise such outcomes.

## Highlights

• Interventional Radiology (IR) and its allied specialties are rapidly expanding; our systematic review suggested that there is a significant gap in the available relevant teaching modules at the undergraduate level globally.
• Lack of exposure to IR teaching opportunities, subsequently affects future motivation towards specialty, as well as decreased awareness and knowledge of IR
• Based on the synthesized evidence, we suggest a framework to trigger an awareness-raising campaign necessary to initiate the branding and creation of IR modules in undergraduate medical education.

## 1 Introduction

Interventional specialities are rapidly expanding, percolating several medical and surgical specialities. The fast-changing technological landscape has inspired the optimisation of imaging modalities which now form the cornerstone of both diagnosis and treatment of various conditions across a number of unrelated specialities. This advancement has permitted the creation of a less invasive and novel role for practitioners in the field of medicine and surgery, that is, the practice of interventional specialities [1]. A classic paradigm is the expansion of interventional cardiology, leading to the displacement and substitution of several extensive cardiothoracic procedures, the latter of which were often associated with higher mortality rates. These procedures are favoured due to having relatively quicker recovery times with shorter hospital stay and decreased cost, accompanied with lower morbidity and mortality [1]. Undoubtedly the number of procedures that are currently being taken over by interventional specialities is increasing rapidly [2]. Interventional Radiology (IR) was granted subspeciality status in the UK in 2010, and the number of IR procedures performed is continually increasing [2].

Despite the increasing applicability of interventional procedures in everyday practice, there is still a significant gap in the teaching available to undergraduate medical students [3]. Currently there are no relevant structured teaching modules in medical school curricula reported; this applies for example to schools both in the USA and China [4].

In most cases, teaching efforts are limited to diagnostic radiology, and such learning outcomes are commonly integrated as part of the basic sciences curriculum [3]. Therefore, isolated focus on IR principles is almost non-existent whilst exposure to practical IR interventions is usually indirect and coincidental. Whilst IR has indeed become a sub-specialisation in radiology with growing relevance to everyday medical practice, reconsideration of its place in medical education has yet to occur. Indeed, several studies confirm lack of knowledge towards those specialities which limits knowledge of several procedures and interventions which are now mainstay in clinical practice. Inevitably, this is expected to lead to inadequately trained doctors, the significance of which becomes greater with time considering the growing relevance of IR procedures in everyday patient management.

A study across three Canadian medical schools concluded that 91% of students requested more radiology teaching [5]. Minimal exposure during medical school impacts interest and motivation toward these specialities, likely due to lack of knowledge of the field [[6], [7], [8], [9], [10]]. This lack of interest towards interventional specialities harbours poor future recruitment potential. In light of the Royal College of Radiologists announcement in 2017, highlighting the need for an additional 222 consultants to meet current staffing targets in acute trusts [2], recruitment and workforce planning is becoming a greater priority for IR.

We performed a systematic review to identify all available learning modules for interventional specialities aimed at the undergraduate level; and secondarily, to quantify their impact on motivation and performance improvement. Based on this we aimed to conclude to a unified Strategy Development Framework for Interventional Radiology (SDFIR) for enhancement of undergraduate learning towards interventional specialities.

## 2 Methods

We followed a prospectively designed protocol which met the Preferred Reporting Items for Systematic Reviews and Meta-Analyses (PRISMA) statement.

### 2.1 Registration

This systematic review has been registered with PROSPERO (Registration: CRD42018110006) and assessed against the AMSTAR2 critical domains.

### 2.2 Selection criteria

Our selection process was strictly limited to our pre-determined PICO criteria, consisting of Population, Interventions, Comparison and Outcomes, respectively. Our chosen population included any undergraduate medical student, while interventions pertained to any teaching module in undergraduate education such as simulation or teaching courses, or any other learning activity relating to interventional specialities. We defined interventional specialities as any of the following: interventional radiology, interventional cardiology, interventional pulmonology, interventional anaesthesiology, interventional vascular surgery. Although there was no actual comparator, we assumed that comparison will be performed between different interventional specialities in the form of a subgroup analysis. Primary outcomes included performance improvement (outcome 1) or motivation towards a career in an interventional speciality (outcome 2).

Studies were included regardless of duration, as long as they reported at least one of our pre-specified outcomes. These studies could be in the form of undergraduate survey responses on interventional specialities or specific courses or teaching modules where objective performance of delegates was assessed. We excluded any studies relating to postgraduate courses or studies that did not report at least one of our preferred outcomes.

### 2.3 Search strategy

Search strategy was designed to meet the PICO strategy. We looked at PubMed and Ovid with no language restriction until 27th December 2017. We also manually searched the references of any included titles. We used the following combination of keywords: (((“interventional radiology” or “interventional cardiology” or “interventional pulmonology” or “interventional pain” or “interventional vascular”)) AND (“simulation based learning” or “medical education” or “teaching” or “training” or “learning” or “assessment” or “course” or “seminar” or “module” or “wet lab” or “dry lab” or “cadaveric”)) AND (“undergraduate*” OR “medical student*” or “undergraduate curricul*” OR “medical school curricul*”)

## 3 Screening of the literature and data extraction

Initial screening of the titles was performed by two independent reviewers (EIE/ZR). A third and fourth independent reviewer confirmed the validity of the extracted data (JH/IT). In the case of conflict, this was resolved by one of the senior authors (MS). A similar strategy was followed for data extraction; we extracted the data using predesigned excel sheets.

## 4 Quality assessment

As we anticipated variation in reported outcomes, we designed a modified set of questions aimed to assess internal and external validity of included studies based on previously published methodology [11].

Internal validity was assessed using the following parameters: study design, recruitment of undergraduate students, ascertainment of the reported outcomes, follow-up of participants and misclassification bias. Study design was classified as prospective or retrospective; recruitment of participants fell in one of the following categories: consecutive, randomised or arbitrary. Moreover, “ascertainment of reported outcomes” was scrutinised based on the choice of assessment tool, such as whether studies used validated performance assessment tools, validated feedback questionnaires or whether assessment was made by independent assessors or a single expert assessor. Follow-up of >80% was considered as adequate; in the case of single point survey, follow-up was considered as the response rate. Lastly, for external validity, we assessed the representativeness of the included population by defining the background of involved students, categorising them as either ‘exclusively motivated to speciality’ or ‘mixed’.

All validity parameters were evaluated and subsequently classified on a risk-of-bias scale, such that when any of the identified parameter was not mentioned in each study's methods, this was considered as “high risk”. If a study was determined to pertain high risk of bias in more than two parameters for internal validity, the study was overall classified as “high risk” for internal validity. The single item (population representativeness) classified each study for high or low risk for external validity.

## 5 Results

**Fig. 1:**
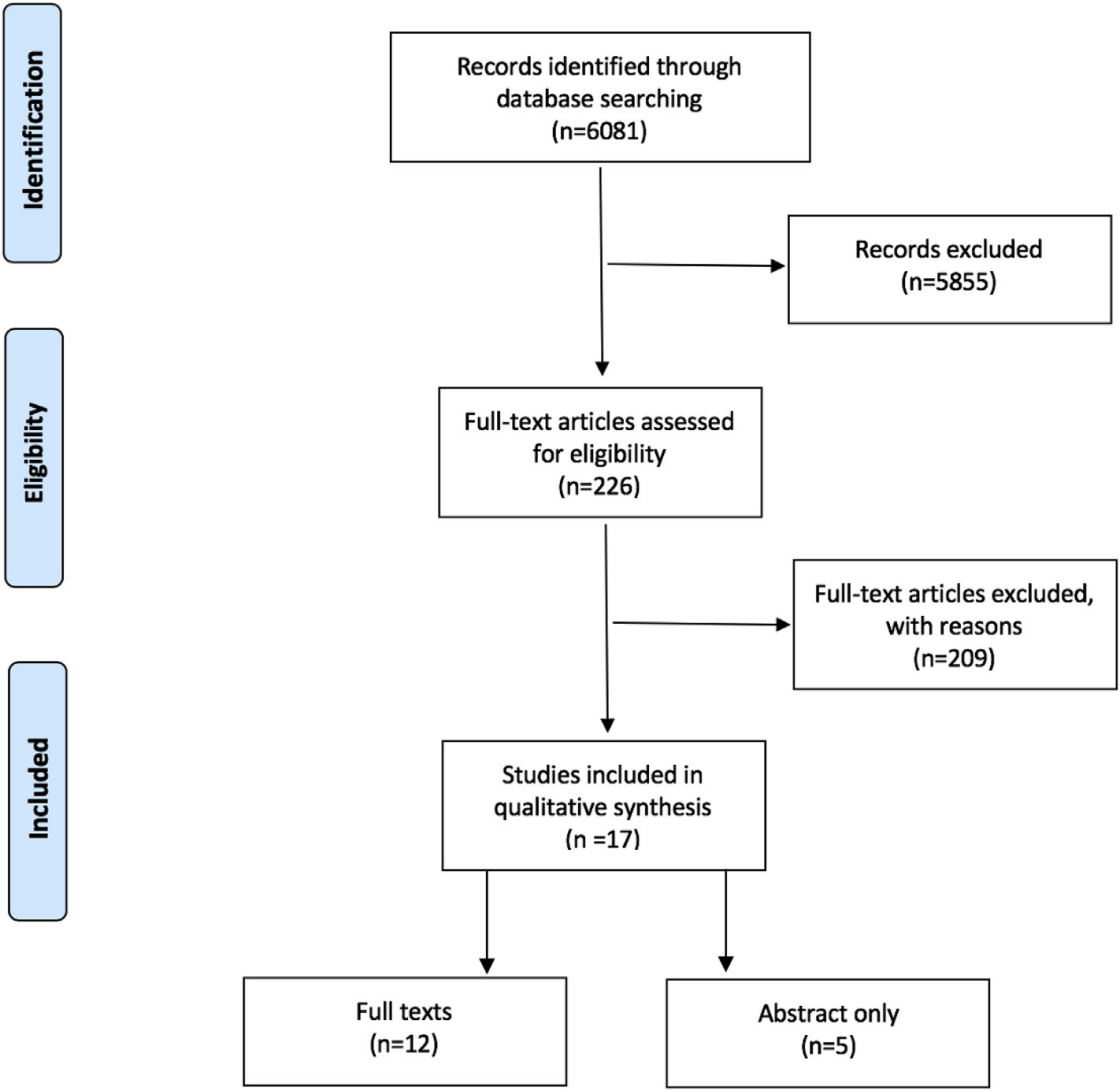
PRISMA diagram.

A total of 6081 titles and abstracts were identified (Fig. 1); we excluded 5855 titles as irrelevant or duplicates. Full text assessments were performed in the remaining 226 abstracts and 17 papers were included in the qualitative synthesis. A total of 3665 students were included across all the studies, except for two studies where no participant number was recorded [12,13]. A total of five were conference abstracts (abstract only) [10,[12], [13], [14], [15]].

### 5.1 Included studies’ characteristics

Table 1 summarises the main characteristics of the included studies such as the scope of the study, the region it was performed in, the number of participants, the speciality and the type of intervention introduced. Detailed characteristics of the included studies are provided in the Appendix.

**Table 1:** Summary of all eligible studies.

Author	Year	Country	Participants	Intervention	Speciality	Scope of Assessment			
						Awareness	Interest	Skills	Knowledge
Lee et al. [19]	2011	USA	52	Elective course	EV Surgery			✓	
Alexander et al. [20]	2015	USA	73	Symposium	IR	✓	✓		✓
Ghatan et al. [21]	2010	USA	64	Lecture	IR	✓	✓		
Shaikh et al. [22]	2016	Ireland	309	Lecture	IR	✓	✓		✓
Brascher et al. [23]	2014	Germany	29 (12 medical students)	Elective course	Anaesthesiology			✓	
Alsafi et al. [18]	2017	UK	51	Survey	IR	✓			✓
DePietro et al. [16]	2017	USA	146	Elective course	IR		✓		✓
Atiiga et al. [6]	2017	UK	220	Survey	IR	✓	✓		✓
Rehman et al. [9]	2016	Pakistan	288	Survey	IR		✓		✓
Commander et al. [17]	2014	USA	845	Survey	IR	✓	✓		✓
O'Malley et al. [7]	2012	Canada	542	Survey	IR	✓	✓		✓
Alshumrani et al. [8]	2013	Saudi Arabia	119	Survey	IR	✓	✓		✓
Coupal et al. [12]	2014	USA	Unavailable	Symposium	IR	✓	✓		
Hanif et al. [13]	2014	USA	Unavailable	E-learning platform	IR				✓
Caci et al. [15]	2014	Sint Maarten	105	Survey	IR/DR	✓			✓
Bunney et al. [14]	2014	USA	329	Survey	IR		✓		✓
Commander et al. [10]	2014	USA	510	Survey	IR		✓		✓
					Total	10	12	3	14

Included studies were conducted between 2010 and 2017, implying that all studies have been carried out in the past 10 years, with 14 studies carried out within the last five years. More than half of the studies were carried out in the USA (9/17), and only two in the UK. Great variation was observed both in the number of participants per study, ranging from 12 to 845 students, as well as the year of study of participants, ranging from second to final years. The intervention length varied from 1 h to 8 weeks, yet many studies failed to include specific details regarding the duration of interventions. A total of 15 studies were themed around IR, one around endovascular surgery and one around image-guided anaesthesiology. Nine out of seventeen studies consisted of surveys sent to undergraduate medical students, focusing on assessing motivation and background knowledge of interventional specialities. The majority of surveys directly questioned the potential need for additional or compulsory IR-specific modules, providing insight into future proposals for undergraduate training improvement. Six studies used a combination of surveys, performance-based tests and feedback and one study was limited to the description of an e-learning platform for IR teaching [13]. Lastly, one study reported a symposium that had been repeated twice in an effort to develop interest in IR [12]. Due to the significant variation in the intervention types and reported outcomes measurements, results could not be unified under a single domain. Nevertheless, Table 1 presents the scope of assessment in each study.

### 5.2 Quality assessment

**Fig. 2:**
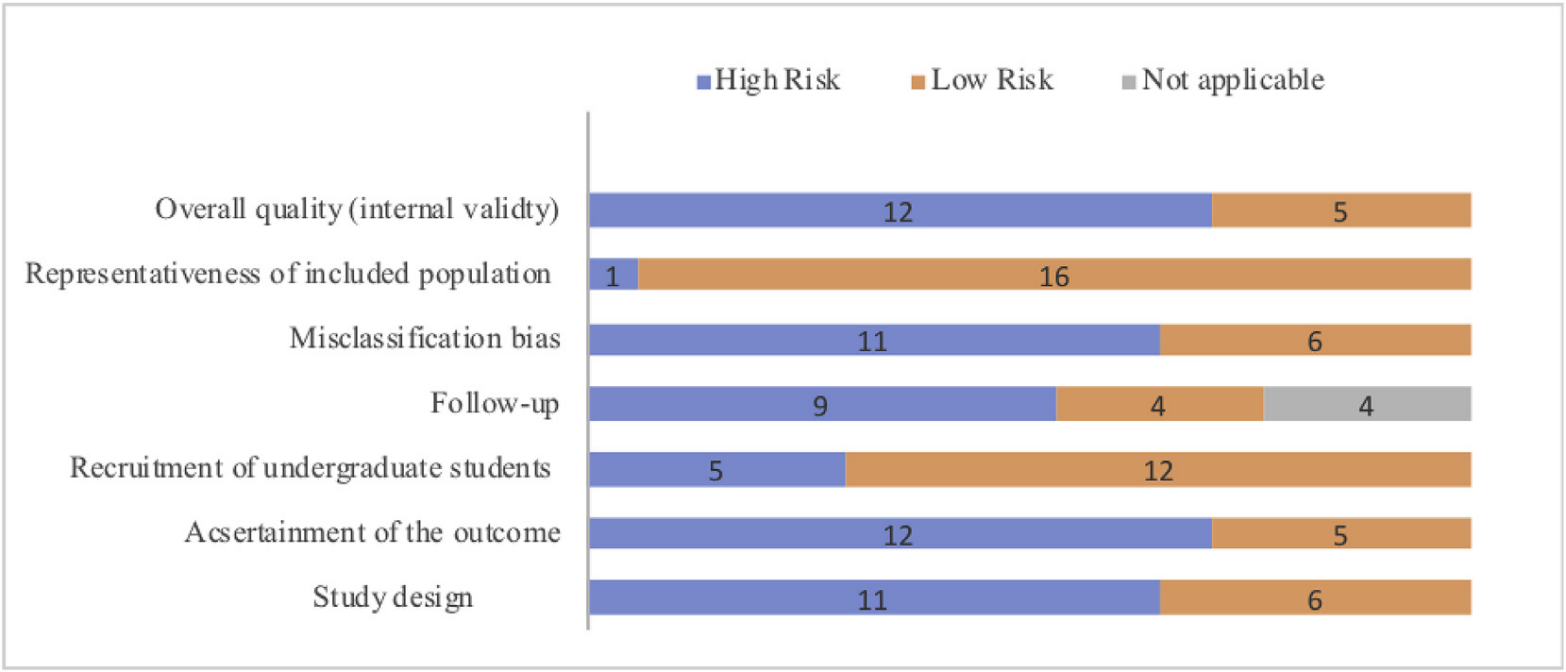
Quality assessment of included studies.

Based on the parameters assessed, 12 studies were classified as “high risk” for internal validity [[6], [7], [8], [9], [10],[12], [13], [14], [15], [16], [17], [18]] and 5 as “low risk” [[19], [20], [21], [22], [23]]. Sixteen studies used a representative population of undergraduate students and were therefore considered as low risk for external validity. Fig. 2 summarises the quality assessment. According to AMSTAR criteria, the overall confidence in the results of the review is moderate.

### 5.3 Reported outcomes and conclusions

Nine out of seventeen studies reported students’ perceptions on interventional specialities, primarily based on surveys or feedback questionnaires (Table 1) [[6], [7], [8], [9], [10],^14^,^15^,^17^,^18^]. Six studies reported performance improvement in the form of structured skills-based assessments or knowledge-based questionnaires on understanding of the speciality [16, [19], [20], [21], [22], [23]]. Six studies assessed the knowledge of students through randomly distributed questionnaires [6,9,10,14,15,18]; another six with questionnaires distributed after learning modules [16,[19], [20], [21], [22], [23]]. Five of the former studies mentioned used a pre and post-intervention survey to test for improvement after the relevant learning modules [16,[19], [20], [21], [22]]. Table 2 describes the outcomes reported in each study, along with the relevant conclusions.

**Table 2:** Outcomes of eligible studies.

Author	Speciality	Duration	Outcomes				Conclusions
			Awareness assessment	Interest assessment	Skill assessment	Knowledge assessment	
Lee et al. [19]	Endovascular Surgery	8 weeks		✓	✓		
				‘Very interested and ‘Interested’ increased to 90%	114% (1.73/5 to 3.7/5) P < 0.001		
Alexander et al. [20]	IR	6 h	✓	✓		✓	Start exposure in preclinical years (94%) or clinical years (6%)
			Awareness very limited	From 58% to 69%, i.e., 19% increase (P = 0.0293)		Non-significant	
Ghatan et al. [21]	IR	1 h	✓	✓			Principles of IR should be known irrespective of career choices (86%)
			‘Significantly improved’ and ‘better’ impressions increased to 75%	19%–33%			
Shaikh et al. [22]	IR	10 h	✓	✓		✓	
			Awareness very limited	60%–73% (P∼0.008)		From 4% (‘good’) and 2% (‘excellent’) to 45%	
Brascher et al. [23]	Anaesthesiology	n/a			✓		
					Decreased procedure time by 53.1% (P = 0.001) and number of corrections (P = 0.014)		
Alsafi et al. [18]	IR	n/a	✓			✓	
			Awareness very limited			Scores in IR (38.4%) lower than non-IR (56.9%) (P < 0.005)	
DePietro et al. [16]	IR	n/a		✓		✓	
				Increased from 24% to 64% (P < 0.05)		Lack of IR knowledge decreased from 73% to 27% (P < 0.001)	
Atiiga et al. [6]	IR	n/a	✓	✓		✓	55.5% felt knowledge of IR training was poor and 81% never received teaching
			Awareness very limited	Only 15% considered a career in IR prior to course		n/a	
Rehman et al. [9]	IR	n/a		✓		✓	81% uninterested or unsure in pursuing a career in IR, mainly due to lack of interest and exposure
				Only 66.7% interested in 2-week rotation in radiology		4% ‘excellent’ knowledge, 53.9% ‘poor’ or nil knowledge	
Commander et al. [17]	IR	n/a	✓	✓		✓	Main reason for lack of interest is the lack of teaching and exposure to IR in medical school
			Awareness very limited	Interest in IR: 54% in MSR vs. 38% in MSE (P < 0.003)		‘Excellent’ in 18% in MSR students vs. 5% of MSE	
O'Malley et al. [7]	IR	4 weeks	✓	✓		✓	Main reasons for lack of interest in IR career is lack of knowledge (48%)
				82% either uninterested or unsure about a career in IR		53% poor knowledge of IR	
Alshumrani et al. [8]	IR	4 weeks	✓	✓		✓	Main reasons for lack of interest in IR is the lack of knowledge (33%)
			Awareness very limited	62% either not or unsure of considering IR as a career		52% poor knowledge of IR	
Coupal et al. [12]	IR	n/a	✓				Symposiums spark interest and awareness of electives in IR
			Reported increase				
Hanif et al. [13]	IR	n/a				✓	Expected to engage interactive learning and create a safe environment for students to explore IR
						n/a	
Caci et al. [15]	IR/Diagnostic Radiology (DR)	n/a	✓			✓	85% recommendation rate, with 46% wanting to see IR as a separate rotation
			Reported increase			from 36% to 85% (P < 0.001).	
Bunney et al. [14]	IR	n/a		✓		✓	
				Interest of 1.8/5 would increase if IR was a primary residency (3.6/5)		Score of 33% in no-rotation vs. 44% after radiology rotation (P < 0.01)	
Commander et al. [10]	IR	n/a		✓		✓	76% would prefer lectures from IR specialists and 69% would be interested in a 2-week IR rotation
						76% had fair or poor knowledge of IR	

### 5.4 Studies involving educational intervention

Five studies reported performance improvement after application of the interventions [16,19,[21], [22], [23]], of which four were statistically significant [16,19,22,23]. One study reported no performance improvement [20], whilst three studies reported a statistically significant increase in students’ motivation [16,19,22]. In two studies there were no performance or motivation outcome results reported [12,13].

### 5.5 Studies involving surveys only

Four studies reported poor exposure to relevant educational opportunities [6,[8], [9], [10]], and six studies reported lack of knowledge relating to basic interventional procedures [[6], [7], [8], [9], [10],^14^]. One study commented on the positive effect of IR placement exposure on the knowledge of and motivation towards the relevant speciality, which was statistically significant [17]. Another study which involved surveys sent to students after a radiology rotation showed a statistically significant increase in knowledge after the rotation [15].

### 5.6 Subgroup analysis for studies focused on interventional radiology

Table 3 summarises the increase in motivation towards a career in IR following exposure as a medical student. Eight studies demonstrated an increase in motivational potential [[6], [7], [8], [9],^16^,^17^,^20^,^22^], four of which reported statistically significant outcomes [16,17,20,22].

**Table 3:** Comparison of interest in IR across 8 studies.

Studies	Interest in a career in interventional medicine (%)		Statistically significant increase in interest? (P < 0.05)
	Pre-exposure to IR (study intervention e.g. lecture, simulation/clinical rotation in radiology or IR)	Post-exposure to IR	
Alexander et al. [20]	58	69	Yes
Alshumrani et al.^a^ [8]	38	–	–
Atiiga et al.^a^ [6]	15	–	–
Commander et al.^b^ [17]	38	54	Yes
DePietro et al. [16]	24	64	Yes
O'Malley et al.^a^ [7]	18	–	–
Rehman et al.^a^ [9]	19	–	–
Shaikh et al. [22]	60	73	Yes

(−) no data available.

a Survey only. Cohort of students with minimal, if any, exposure to interventional specialities.

b Pre-exposure group are students from a medical school with no radiology elective. Post-exposure group are students who have undergone an attachment in radiology.

## 6 Discussion

### 6.1 Is IR really neglected?

Our study has confirmed that interventional specialities such as IR are in growing demand, yet their presence in undergraduate medical education is at best in its infancy [24]. Indeed, most studies have underlined the limited exposure and teaching available in IR at the undergraduate level [15,17,18], with national surveys echoing these findings by revealing that only 0.4% of students receive official teaching from a dedicated IR syllabus [25]. Our review has also highlighted poor knowledge and understanding of such specialities, which likely hinders medical students’ motivation towards a career in them.

### 6.2 What can be done?

The majority of interventions included in this review, such as practical workshops, lectures or informative surveys, increased students' interest, knowledge, skills or awareness towards these specialities. Similarly, rotating on a clinical placement in radiology does seem to ignite interest and motivation to pursue a relevant career [10,14,15]. This in turn is expected to be beneficial for future recruitment practices in an effort to cover any staffing shortages which have recently started to arise in these specialities. Implementing early exposure to such activities at medical school is crucial. Several studies in other medical disciplines have clearly demonstrated that students’ career choices are often strongly influenced by experiences early-on in their undergraduate training [[25], [26], [27], [28]].

Generally, studies included in this review suggest that simple measures, such as didactic lectures can increase students’ knowledge of and interest in IR. Simulation-based learning (SBL) sessions also exert a strong effect, especially when evaluating their use in the acquisition and improvement of image-guided skills such as cannulation and other tasks which are easily reproducible in simulation environments. Interestingly, the majority of interventions, such as courses and symposiums, were completed over a few hours or a handful of weeks, yet the outcomes were overwhelmingly positive. We therefore suggest a balanced introduction of such SBL modules, tailored with traditional teaching methods such as didactic introductory lectures and case-based discussions. The Royal College of Radiologists (RCR) has an undergraduate radiology curriculum which offers particular learning outcomes, including a list of essential core competencies that a medical student should acquire prior to progession to foundation training programme [29]. Whilst the majority of learning modules evaluated in this systematic review were from USA-based studies, their simplicity and reproducibility suggests high external validity, and generalisability internationally.

### 6.3 Why is this important?

Deciding whether IR-related teaching deserves a stand-alone module in medical education is a crucial question to address. Few medical schools in Europe and the USA offer a clinical attachment in radiology as part of their curriculum and making a case for this change will be challenging. A previous survey sent out to 675 final year medical students in Dublin showed that 65% of students had not completed a radiology elective [28]. Despite the local character of this survey, this could be a representation of sparce opportunities to be exposed in such modules. Similar findings were reported by a Spanish survey, which underlined that local medical students had limited exposure to and knowledge of IR; of which 98% of clinical and 100% of preclinical students would like to be exposed to IR in their curriculum [30]. Similar findings were reported from a recent Australian cross-sectional survey [31]. On the other hand, many students agreed that IR should be a separate clinical rotation [6,7,9,10,20,21]. Although a very small proportion of students seem to be interested in IR, it was agreed by 86% of respondants that the principles of IR should be known irrespective of final career choice [21]. This is in keeping with some studies’ conclusions that students strongly favour additional teaching in IR; by means of lectures, clinical attachments and mentoring.

The Royal College of Radiologists in the UK has reported a shortage in the number of applicants who are considering the speciality, whilst demand for and reliance on interventional procedures continues to grow [2]. This demand for interventional procedures is driven by various factors, including in some cases improved outcomes and increased patient safety compared to the equivalent surgical procedure, or the possibility of reducing length of hospital stay which eases cost and bed-occupancy pressures on hospitals and health systems. There have been several studies on factors which influence medical students’ speciality choice. 58% of students from one study carried out in the Middle East thought that the reputation of speciality was an important factor when considering to pursue it [32]. An additional study showed that successful recruitment of students to a particular speciality correlated with a stronger undergraduate curriculum in the specific field alongside stronger academic reputation [33]. This was seen to be poor in the studies from this review with very few learning modules, if any, in IR. However, it is important to attract students and inform them from an early stage.

### 6.4 A framework for future interventions

A recent letter from Ireland underlines the need for taking action and initiate formal action to include IR modules in the undergraduate curricula [ref]. Our review has also highlighted the widespread evidence available to similarly suggest that undergraduate medical education suffers from a significant lack of IR exposure globally. Whilst the introduction of IR principles in medical education is appealing, the absence of systematic approaches renders effective incorporation of IR in existing curricula difficult.

**Fig. 3:**
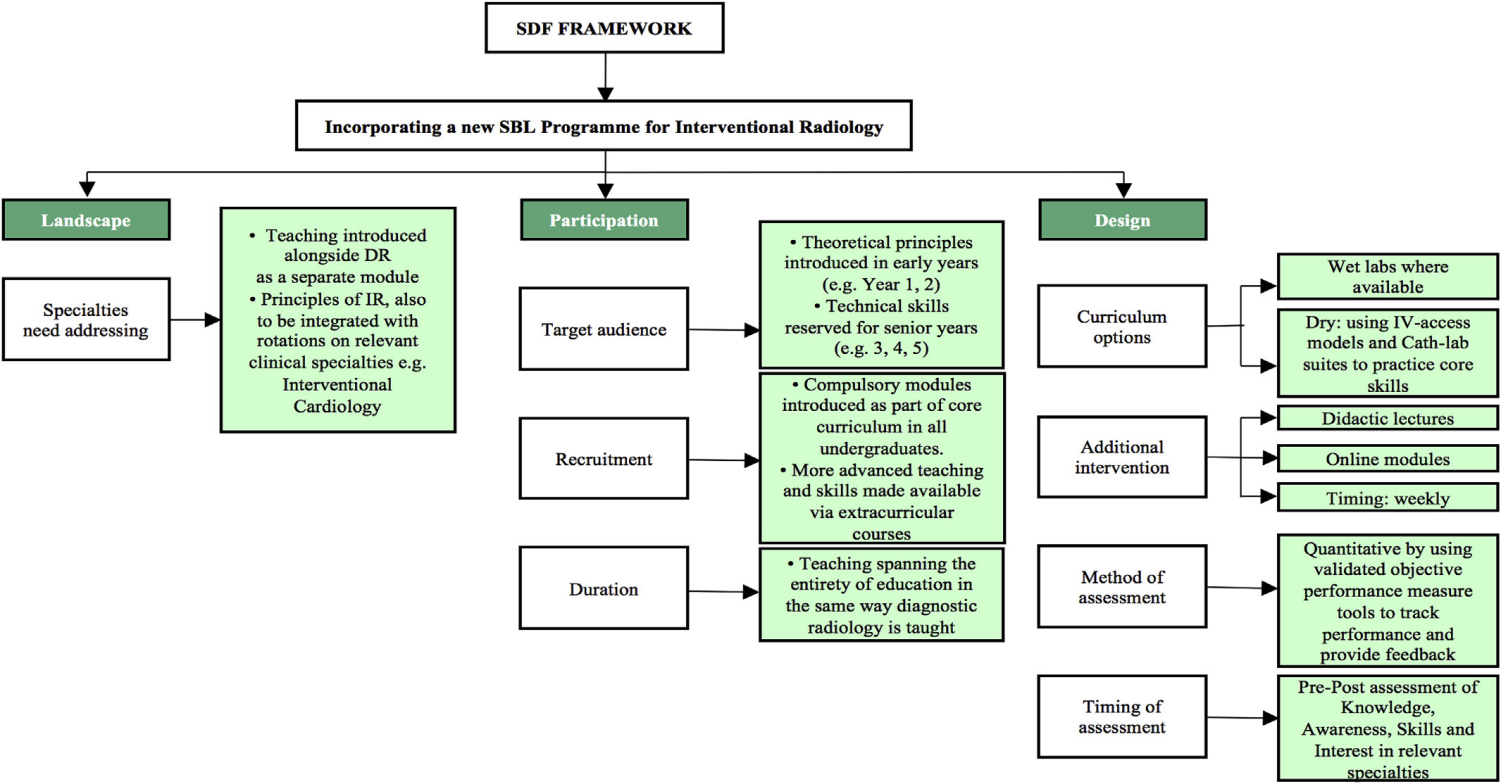
Sdfir.

In our previous study we proposed an SDF framework for the development of SBL courses in undergraduate surgical education [34]. We hereby propose an SDF dedicated to interventional radiology and allied specialities (Fig. 3). Designing the SDFIR upon the principles of surgical education might at first appear controversial, however, given the significant resemblance and proximity of IR to surgical principles such as manual dexterity and the soft skills necessary in leading teams in theatres, we believe there is sufficient congruity between these two areas of medicine to make our proposed model a valid one for undergraduate education. We recognise that our proposed framework is amenable to criticism and further improvement. For example, we predict that its complete implementation will at first be costly and may interfere with other endeavours such as optimising undergraduate surgical training. Therefore, the chief aim of this framework is to act as a call to action for a much-needed awareness-raising campaign to kick-start the implementation of IR modules in undergraduate education.

## 7 Limitations

We recognise a series of limitations in our systematic review. Only 17 studies qualified to be included in this SR which reflects a small number. There is also significant heterogeneity of reported outcomes and relevant results; this can be attributed to a significant variation in the nature or size of audience including year of studies, country of origin, number of participants etc. Additionally, such specialities are newly implemented and students are hesitant to engage. Furthermore, we acknowledge that not all curricula and teaching initiatives are peer-reviewed and published, hence likely underestimating the true scope of interventional teaching initiatives. Based on our quality assessment 12/17 studies were deemed “high risk” for internal validity; only 12 out of 17 achieved adequate ascertainment of the reported outcomes and 11/17 had misclassification bias. The majority of studies were based on student perspective and subjective judgement of performance which did not use validated scales to measure reported outcomes. Study design was retrospective in 11/17 cases. On the other hand, only one included study had non-representative population for our inclusion criteria.

## 8 Conclusion

This systematic review has highlighted the lack of available teaching in IR as an existing problem, potentially attributing to difficulties with recruitment to speciality. A general trend across all studies is evident; knowledge and interest are poor in greater than half of students due to lack of knowledge and exposure. This can result in poor technical knowledge and a lack of motivation towards a career in interventional medicine. However, it is also evident that simple changes could be implemented to increase interest, knowledge and insight of medical students. Based on our previous research, we suggest a novel reproducible framework to set up relevant teaching modules, either as part of the medical curriculum, or more generally as part of any teaching activity, including course or placement.

## Ethical approval

Not applicable.

## Funding

Funded in the form of scholarships as part of the ESMSC Medical Education Research Group (eMERG), by the Experimental, Educational and Research Center ELPEN.

## Author contribution

Michail Sideris (MS) and Apostolos Papalois (AP) conceived the study; MS/AP are the eMERG Academic Leads who decided to fund principal authors in the form of scholarships to perform this study. John Hanrahan (JH) has contributed to the conception of the protocol and designed the search strategy and data extraction sheet; JH has performed primary editing and checked integrity of language and accuracy of presented results. MS has edited the manuscript and holds responsibility for the CRD42018110006 protocol. Elif Iliria Emin (EIE) is the principal (first) author who screened the literature, extracted the data, drafted the manuscript and incorporated senior authors’ input. Zeinab Ruhomauly screened literature and extracted data and contributed to tables/figures of the manuscript; ZR has performed philological editing of the manuscript. Iakovos Theodoulou (IT) has confirmed validity of the data and performed major editing of the discussion and results; IT performed minor edits in the introduction and methods. ZR and IT are equal contributors, as second authors, with major input in the final version of this systematic review. Marios Nicolaides and Nikolaos Staikoglou have contributed in data presentation and editing of the manuscript. Narayanan Thulasidasan is a Consultant in Interventional Radiology who offered senior input in the final design and editing of the discussion. All authors have edited and approved final version of the manuscript.

## Conflicts of interest

None declared.

## Research registration number

PROSPERO registration number: CRD42018110006.

## Guarantor

Michail Sideris is the guarantor of this work.

## Provenance and peer review

Not commissioned, externally peer reviewed.

## Figures and Tables

**Table:** 

Author	Year	Country	Participants (number)	Intervention	Speciality	Scope
Lee et al.	2011	USA	52	Elective course	Endovascular	Long-term follow-up which assessed the ability of a simulation-based curriculum to improve continued interest in speciality
Alexander et al.	2015	USA	73	Symposium	Vascular IR	Assessed the ability of a symposium in educating medical students about IR and increasing interest
Ghatan et al.	2010	USA	64	Lecture	IR	Examined awareness and perceptions of IR amongst medical students; in order to encourage the integration of IR into the medical curriculum
Shaikh et al.	2016	Ireland	309	Lecture	IR	Determined the impact of a new IR curriculum on perception, knowledge and interest of medical students
Brascher et al.	2014	Germany	29 (12 medical students)	Elective course	Ultrasound (US)-guided procedures	Developed a training curriculum to enable beginner medical students and anaesthesiologists to learn relevant skills in US-guided procedures
Alsafi et al.	2017	UK	51	Survey	IR	Evaluated final-year medical students' perception of IR by assessing their knowledge of some common procedures compared to minimally invasive non-IR procedures
DePietro et al.	2017	USA	146	Elective course	IR	Compared medical student knowledge of and interest in IR before and after the integration of an IR lecture series
Atiiga et al.	2017	UK	220	Survey	IR	Investigated awareness, knowledge, and interests in IR among final year medical students
Rehman et al.	2016	Pakistan	288	Survey	IR	Evaluated knowledge of and interest in IR among medical students
Commander et al.	2014	USA	845	Survey	IR	Determined difference in knowledge of IR between medical students in preclinical years compared with clinical years at two medical schools. Compared awareness of IR based on the available curriculum: one with compulsory radiology education and one without
O'Malley et al.	2012	Canada	542	Survey	IR	Assessed awareness and level of exposure of IR and how IR can be better introduced to medical students.
Alshumrani et al.	2013	Saudi Arabia	119	Survey	IR	Assessed awareness of IR among final-year medical students and medical interns
Coupal et al.	2014	USA	Not reported.	Symposium	IR	Assessed a symposium in 2012 and shared lessons learned and delineate improvements in the enhanced newer version in 2013 (IR 2.0)
Hanif et al.	2014	USA	Not reported.	E-learning platform	IR	Description of a developed e-learning website for IR; consisting of teaching modules and assessments which can be reviewed by program directors to provide feedback to students
Caci et al.	2014	Sint Maarten	105	Survey	IR/DR	Evaluated awareness of the new dual IR/DR certificate with focus on effective integration of an IR core rotation in medical student curriculum
Bunney et al.	2014	USA	329	Survey	IR	Assessed awareness of basic IR procedures and interest in the speciality.
Commander et al.	2014	USA	510	Survey	IR	Assessed knowledge and awareness of IR amongst first, second and third year medical students at a single institution
Mulligan et al.	2013	USA	55 (45 medical students)	Symposium	IR	Introduced medical students IR and motivate students to seek out research and future career opportunities within the field
